# Outcomes and Cost of Major Liver Resection Using Combined LigaSure and Stapler: A Propensity Score Matching Study

**DOI:** 10.3390/jcm14113892

**Published:** 2025-06-01

**Authors:** Sepehr Abbasi Dezfouli, Arash Dooghaie Moghadam, Nastaran Sabetkish, Elias Khajeh, Ali Ramouz, Ali Majlesara, Markus Mieth, De Hua Chang, Mohammad Golriz, Arianeb Mehrabi

**Affiliations:** 1 Department of General, Visceral, and Transplantation Surgery, Heidelberg University Hospital, 69120 Heidelberg, Germany; sepehr.abbasidezfouli@med.uni-heidelberg.de (S.A.D.); arash.moghadam@umm.de (A.D.M.); nastaran.sabetkish@med.uni-heidelberg.de (N.S.); elias.khajeh@med.uni-heidelberg.de (E.K.); ali.ramouz@med.uni-heidelberg.de (A.R.); ali.majlesara@med.uni-heidelberg.de (A.M.); markus.mieth@med.uni-heidelberg.de (M.M.); mohamad_golriz@yahoo.com (M.G.); 2 Liver Cancer Center Heidelberg (LCCH), Heidelberg University Hospital, 69120 Heidelberg, Germany; 3 Department of Diagnostic and Interventional Radiology, Heidelberg University Hospital, 69120 Heidelberg, Germany

**Keywords:** hepatectomy, LigaSure, liver transection, major liver resection, stapler

## Abstract

**Background:** Bile leakage remains a significant challenge following major liver resection, with potential for improvement depending on the transection technique used. In this study, we aimed to evaluate the effectiveness of our hybrid resection technique—utilizing both LigaSure and stapler devices—in reducing bile leakage after major liver resection compared to our conventional stapler-only technique. As a secondary aim, we compared overall morbidity, costs, and reimbursements. **Method:** Patients who underwent major hepatectomy without biliary reconstruction using either the hybrid or stapler technique between August 2014 and December 2021 were included in the study. Propensity score matching was performed using a one-to-two algorithm. Perioperative data, bile leakage rates, and cost and reimbursement information based on the diagnosis-related group (DRG) system were analyzed. **Results:** In total, data from 492 patients were evaluated (hybrid = 152; stapler = 340). After one-to-two propensity score matching, the operation time was significantly longer in the hybrid group (*p* = 0.005). A cost analysis showed no significant difference in total operative costs between the two techniques (*p* = 0.092). However, the hybrid group had a significantly lower rate of bile leakage (*p* = 0.002), as well as shorter intensive care unit (ICU) and overall hospital stays (*p* = 0.034 and *p* = 0.007, respectively). Consequently, ICU and ward costs were significantly lower in the hybrid group (*p* = 0.024 and *p* = 0.014, respectively) compared to the stapler group. The financial difference calculated as DRG reimbursement minus costs was two-fold higher in the hybrid group (*p* = 0.02). **Conclusions:** Although the hybrid technique resulted in a longer operating time, it proved superior to the stapler technique in reducing postoperative bile leakage and shortening ICU and hospital stays. Furthermore, the use of the hybrid technique was more cost efficient and resulted in a greater positive financial margin.

## 1. Introduction

Major liver resection remains the standard of care for managing a wide range of benign and malignant tumors, particularly those that are bilobar or large in size [1,2]. Advances in perioperative diagnostic tools, imaging techniques, and therapeutic strategies have contributed to an increased number of patients eligible for major liver resections. Despite these improvements, the procedure continues to be associated with significant perioperative morbidity, with complication rates reported between 20% and 50% [3,4].

Among these complications, bile leakage stands out as one of the most frequent and technically challenging issues, especially following major resections [5]. Bile leakage prolongs hospital stays, increases healthcare costs, and raises the risk of reoperations and mortality [6,7,8]. Bile leakage may arise from different anatomical sites and mechanisms. In cases involving biliary duct reconstructions, leakage often occurs at the site of the anastomosis. Such anastomotic leaks are typically influenced by factors related to the anastomosis technique, including the suture method, anastomotic tension, and the diameter of the bile duct [9,10]. In contrast, in liver resections without biliary reconstruction, bile leakage originates from the hepatic transection plane. These leaks are primarily related to the surgical approach and the type of devices employed for parenchymal transection and hemostasis [11]. Given the technical nature of bile leakage in most cases, there is potential for reducing its incidence through the refinement of surgical techniques and the integration of advanced transection tools. Therefore, optimizing the choice of devices and methods used during liver transection may play a crucial role in minimizing this complication and improving overall surgical outcomes.

In the last three decades, many liver resection techniques have been introduced to improve postoperative morbidity [12,13,14]. However, none of these techniques have shown better outcomes than the standard clamp-crushing method, which remains the method of choice in many referral hepatopancreaticobiliary centers [15], although significant controversies still exist among authors [16]. In 2001, the stapler method was introduced in our center [17]. Previously, two randomized controlled trials were conducted to compare surgical outcomes and intraoperative blood loss between the stapler versus clamp-crushing and stapler versus LigaSure-only transection methods. It was found that the stapler technique had similar outcomes to the clamp-crushing technique [18] and that the stapler technique had a shorter operating time and lower intraoperative blood loss than the LigaSure-only transection technique [19]. However, a tendency toward lower bile leakage was observed with the LigaSure transection compared to the stapler technique (25% in the LigaSure group versus 35% in the stapler group).

In the following years, a hybrid method using both LigaSure and stapler devices was developed and became increasingly popular. The advantage was that LigaSure appeared to be a safe and efficient device for sealing small bile ducts and vessels, while larger ducts and vessels were sealed with the stapler, combining the benefits of both methods (better parenchymal sealing and faster operation). However, the outcomes of this method had not been evaluated until now. In particular, the additional costs and burden associated with using these devices has never been assessed.

In this study, bile leakage and other surgical outcomes were compared following liver transection using the hybrid and stapler techniques in a matched population of patients who underwent major liver resection. Since both methods involved the use of expensive surgical devices, the intraoperative and postoperative costs, as well as the reimbursements associated with the two techniques, were compared.

## 2. Methods

### 2.1. Study Design

Data from patients who underwent major liver resection using either the hybrid technique or the stapler technique between August 2014 and December 2021 were extracted from a prospectively maintained database. Major liver resection was defined as the resection of more than three anatomical segments based on the Brisbane 2000 Terminology of Liver Anatomy and Resections [20]. Patients younger than 18 years and those undergoing emergency liver surgery, liver resection with biliary reconstruction, vascular reconstruction, or repeated liver resection were excluded. The study protocol was approved by the regional ethics committee of the medical faculty (approval number: S-754/2018) and was conducted in accordance with good clinical practice guidelines and the Declaration of Helsinki.

### 2.2. Surgical Methods

In the hybrid method, the liver capsule was cauterized, and the liver parenchyma was carefully crushed using the LigaSure device (Medtronic plc, Dublin, Ireland) without the sealing function in a stepwise fashion. In each step, visible structures, including the portal triad and hepatic veins (<3 mm), were sealed with the LigaSure device. Bile ducts and vascular structures larger than 3 mm in diameter were clipped. The main bile ducts and vessels in the liver hilum and the main hepatic veins were divided using an Autosuture Endo GIATM Universal Stapler and Endo GIATM Universal Angulating 45 mm Loading Units with 2.5 mm staples (Covidien, Mansfield, MA, USA). An intraoperative drain was placed on the transection plane at the end of the operation.

In the stapler method, a large non-curved vascular clamp was used to compress the liver parenchyma, as previously described [19]. The liver was transected, and the hepatic veins and portal triad were divided using an Autosuture Endo GIATM Universal Stapler and Endo GIATM Universal Straight 60 mm loading units with 2.5 mm staples. An intraoperative drain was placed on the transection plane at the end of the operation.

### 2.3. Patient Data Collection and Measurements

#### 2.3.1. Preoperative Evaluations

Patients’ demographic and clinical data, including age, gender, Body Mass Index (BMI), American Society of Anesthesiologists (ASA) class, indication for resections, and perioperative chemo/radiotherapy were recorded.

#### 2.3.2. Intraoperative Evaluations

The extent of liver resection was documented. Intraoperative blood loss was measured according to the amount of blood absorbed by the gauze and the amount of blood collected in the suction bottle. The amount of blood transfused was also recorded. Operation time was defined as the time between the skin incision and the end of the surgical procedure.

#### 2.3.3. Postoperative Evaluations

Drain fluid was routinely analyzed for bilirubin, and the drains were removed after the third postoperative day if no bleeding or bile leakage was detected. Bile leakage and bleeding were assessed according to the definition of the International Study Group of Liver Surgery (ISGLS) [21,22]. The relaparotomy rate was evaluated, and other surgical complications were recorded and classified based on the Clavien–Dindo classification [23]. The major complication rate was reported as all clinically relevant complications (Clavien–Dindo grade III or higher). The durations of intensive care unit (ICU) and hospital stays were recorded. The recurrence rate was also recorded. Postoperative mortality was defined as all-cause death within 90 days of surgery.

#### 2.3.4. Cost and Reimbursement Evaluations

Total operative costs included operating room costs (including salaries and operating room maintenance) and surgical device costs. Operating room costs were calculated as the cost per minute of operating time multiplied by the duration of the procedure in minutes. To estimate final operating room costs, we used the calculation described by Childers et al. [24]. The device costs were determined by adding the number of staplers to the cost of the stapler instrument. In the hybrid group, the cost of the LigaSure device was also added.

Postoperative costs comprised the cost of the ICU and hospital ward accommodations. The finance department at our hospital calculated the average daily cost of the ICU and hospital ward. These costs included hospitalization, medications, and hospital care. The total ICU cost for each patient was calculated by multiplying the duration of ICU stay by the average daily ICU cost. Total ward costs were calculated by multiplying the duration of hospital ward stay by the average daily ward cost. Total hospital costs were calculated according to the total surgical and postoperative costs of each patient.

Diagnosis-related group (DRG) reimbursement was the total amount reimbursed for the entire index hospital admission, including complication-related costs. This was calculated using German diagnosis-related groups (G-DRGs) [25]. The financial difference was the difference between the DRG reimbursement and total hospital costs.

### 2.4. Statistical Analysis

To avoid selection bias between the two groups as a consequence of confounders, propensity scores were calculated for all patients on the basis of five relevant clinical variables that could influence the final outcome of the comparison. These variables included age, sex, BMI, ASA, and final diagnosis. Finally, patients were selected using a one-to-two algorithm for matching without replacement based on a width of 0.2 of the standard deviation of the propensity scores. Propensity score calculation and selection of the matched cohort was performed using R (R Foundation for Statistical Computing, Vienna, Austria) version 4.2.3. SPSS software (version 25, SPSS Inc., Chicago, IL, USA) was used for the remaining data analysis. The Shapiro–Wilk test was applied to evaluate the normality of data in frequentist statistics. Continuous variables were presented as mean ± standard deviation (SD) or standard error (SE), and categorical variables were presented as frequency distributions. The independent *t*-test or Mann–Whitney U test was used to analyze continuous data. Categorical data were analyzed using either Chi-square or Fisher’s exact test. A two-sided *p* value of <0.05 was considered significant.

## 3. Results

### 3.1. Demographic and Preoperative Data

Between August 2014 and December 2021, 492 patients underwent major liver resection using the hybrid technique (N = 152) or stapler technique (N = 340). Table 1 and Table S1 show the preoperative and postoperative characteristics of these patients. The mean age was 57.7 ± 15.9 years, and 54.7% of the patients were male. The average BMI was 25.7 ± 4.7. Regarding ASA classification, most patients were categorized as ASA II (48.8%) or ASA III (45.7%), while fewer were ASA I (4.5%) or ASA IV (1.0%). The most common diagnosis was classified as “Others” (71.7%), followed by cholangiocellular carcinoma (CCC, 18.5%) and hepatocellular carcinoma (HCC, 9.8%). Preoperative chemo- or radiotherapy was administered to 44.5% of the patients.

Before matching, there were no significant differences in age, gender, BMI, ASA class, diagnosis, and history of chemo/radiotherapy between the two groups. The average age was 56.42 ± 13.5 years in the hybrid group and 57.98 ± 11.4 years in the stapler group (*p* = 0.23). The gender distribution was similar, with 50.0% male in the hybrid group and 56.5% male in the stapler group (*p* = 0.183). BMI was also comparable: 25.47 ± 4.7 in the hybrid group and 25.81 ± 4.67 in the stapler group (*p* = 0.338). The ASA classification showed no significant difference, with most patients in both groups classified as ASA II or III. The most common diagnosis was “Others” (72.4% in the hybrid group and 71.5% in the stapler group), followed by HCC and CCC. The difference in diagnoses was not statistically significant (*p* = 0.622). A similar percentage of patients in both groups received preoperative chemo/radiotherapy (*p* = 0.360).

The patients’ characteristics after matching are shown in Table 2. After propensity score matching, 152 patients in the hybrid group were matched to 304 patients in the stapler group for further analysis. There were no significant differences in age, gender, BMI, ASA class, diagnosis, or history of chemo/radiotherapy between the two groups: the mean age was 59.2 ± 14.8 years in the hybrid group and 58.4 ± 14.8 years in the stapler group (*p* = 0.564). Male patients comprised 50.0% and 56.5% of the groups, respectively (*p* = 0.183). BMI was comparable (25.4 ± 4.9 vs. 25.8 ± 4.8; *p* = 0.402). ASA classification was similarly distributed, with most patients in ASA II or III, and no significant difference (*p* = 0.550). Diagnoses—including HCC (7.9% vs. 10.5%), CCC (19.7% vs. 17.4%), and others (72.4% vs. 72.0%)—were evenly distributed (*p* = 0.599). Preoperative chemo- or radiotherapy was administered to 41.4% of hybrid and 45.4% of stapler patients (*p* = 0.484).

### 3.2. Intraoperative Data

Table 3 presents a comparison of the intraoperative data between the hybrid and stapler groups. There were no significant differences in the type of hepatectomy performed, with right hemihepatectomy performed in 75.7% of patients in both groups (*p* = 1.000) and left hemihepatectomy in 24.3% (*p* = 1.000). Extended hepatectomy was more common on the left side in both groups, with no significant difference (*p* = 0.385). Regarding blood loss, the mean blood loss was 1146.2 ± 821.5 mL in the hybrid group and 1037.6 ± 743.2 mL in the stapler group, showing no significant difference (*p* = 0.170). Similarly, there was no significant difference in the percentage of patients requiring blood transfusion (15.8% in the hybrid group vs. 16.8% in the stapler group, *p* = 0.894) or the amount of blood transfused (mean ± SD: 0.66 ± 2.59 units in the hybrid group vs. 0.33 ± 1.01 units in the stapler group, *p* = 0.260).

However, the hybrid group had a significantly longer operation time (224.8 ± 83.7 min vs. 202.6 ± 67.6 min in the stapler group, *p* = 0.005). The median number of staplers used was significantly higher in the stapler group (11 vs. 4 in the hybrid group, *p* < 0.001).

### 3.3. Postoperative Data

Table 4 compares postoperative data between the hybrid and stapler groups. The hybrid group had significantly lower bile leakage rates (13.2%) compared to the stapler group (25.7%, *p* = 0.002). The incidence of bleeding was also lower in the hybrid group (0.7%) compared to the stapler group (3.9%), although this difference was not statistically significant (*p* = 0.069). The rates of relaparotomy (11.8% in both groups, *p* = 0.566) and major complications (29.6% in the hybrid group vs. 35.2% in the stapler group, *p* = 0.248) were similar between the two groups. Postoperative recovery was faster in the hybrid group, with a significantly shorter ICU stay (5.7 ± 5.3 days vs. 7.6 ± 5.9 days, *p* = 0.034) and shorter hospital stay (14.9 ± 12.7 days vs. 19.1 ± 17.2 days, *p* = 0.007). Mortality rates were similar in both groups (5.3% in the hybrid group vs. 3.9% in the stapler group, *p* = 0.628).

### 3.4. Cost and Reimbursement Outcomes

The results of the cost analysis are presented in Table 5. Regarding surgical device costs, the number of staplers used was significantly different between the two groups (hybrid group: 4 staplers; stapler group: 11 staplers; *p* < 0.001). Surgical device costs were significantly lower in the hybrid group than in the stapler group (hybrid group: EUR 1149 ± 6; stapler group: EUR 1464 ± 6; *p* < 0.001). Operating room costs were significantly higher in the hybrid group (EUR 7417 ± 224 versus EUR 6663 ± 129; *p* = 0.004). The total operative costs were similar between the groups (EUR 8567 ± 224 in the hybrid group versus EUR 8128 ± 128 in the stapler group; *p* = 0.061).

The hybrid group had significantly lower surgical device costs compared to the stapler group (EUR 1149.9 ± 6.6 vs. EUR 1464.9 ± 6.1, *p* < 0.001). Despite higher operating room costs in the hybrid group (EUR 7417.6 ± 224.1 vs. EUR 6663.8 ± 129.6, *p* = 0.004), the total operative costs did not significantly differ between groups (*p* = 0.092).

The total ICU costs were significantly lower in the hybrid group (EUR 7269.3 ± 760.9 vs. EUR 9979.4 ± 918.2, *p* = 0.024), as were total ward costs (EUR 4677.1 ± 385.8 vs. EUR 6044.2 ± 341.5, *p* = 0.014). Consequently, the total hospital costs were lower in the hybrid group, though the difference narrowly missed statistical significance (EUR 18,170.6 ± 737.1 vs. EUR 20,245.9 ± 768.2, *p* = 0.052).

Diagnosis-related reimbursements were comparable between the groups (*p* = 0.740). However, the financial difference (reimbursement minus hospital costs) was significantly greater in the hybrid group (EUR 4898.9 ± 1532.7 vs. EUR 2271.2 ± 1159.8, *p* = 0.020), indicating higher cost efficiency.

## 4. Discussion

In this high-volume study, we compared surgical complications and hospital costs following major liver resection using the hybrid and stapler techniques in matched patients through propensity score matching. We observed a significantly lower incidence of postoperative bile leakage with the hybrid method, as well as shorter ICU and hospital stays. After calculating the costs and reimbursements, the hybrid method proved to be more financially efficient.

The lower postoperative bile leakage observed with the hybrid technique compared with the stapler technique may be explained by the different parenchymal transection accuracies of these two techniques. In the hybrid technique, transection is performed after the liver parenchyma is crushed, which reveals the relevant biliary and vascular structures. This allows the surgeon to ligate the larger structures using metal clips or hemoclips. In contrast, transection with the stapler technique is more “blind.” Therefore, clamp crushing during the hybrid technique offers higher accuracy than stapler transection. On the other hand, this method is faster than clamp crushing since the small biliary and vascular structures are transected directly without additional ligation.

Which transection method is best remains controversial, and different methods are established in various hepatopancreaticobiliary centers. The most commonly used transection method in our center has been stapler transection, which demonstrated less intraoperative blood loss and shorter operation times compared to LigaSure-only transection in a previous study conducted at our institution [18]. However, in the previous study, stapler transection was compared to LigaSure-only transection. Moreover, the study did not exclude patients who underwent biliary reconstruction, introducing bias since the reported bile leakage may not have originated solely from the transection plane. Additionally, both minor and major liver resections were included. In the present study, we addressed these limitations by excluding patients who underwent minor resections or required biliary reconstruction.

Although controversial, some studies have shown that the LigaSure transection results in less intraoperative blood loss than the traditional crush-clamping technique [26,27,28]. In an RCT by Ikeda et al., a combined parenchymal resection method that included clamp crushing and LigaSure was compared with the conventional clamp-crushing method in 165 patients. The primary endpoint was liver transection time, and similar results were reported in both groups. Regarding bile leakage, no significant difference was observed between the two methods (6.7% in the LigaSure group versus 11% in the clamp-crushing group) [28]. In another more recent RCT, Ichida et al. [29] evaluated liver resection results using a combination of clamp-crushing and energy devices (LigaSure or the ultrasonically activated device) in 116 patients, with the primary endpoint being blood loss during liver transection. In this study, a slight but significantly reduced blood loss was observed in the LigaSure group compared to the clamp-crushing method. Other complications were similar in both groups. Regarding bile leakage, there was an insignificant trend toward less bile leakage in the LigaSure group (4.1% in the LigaSure group versus 6.1% in the clamp-crushing group, *p* = 0.371). However, both studies failed to show a significant difference in bile leakage because the studies were not powered to assess bile leakage. Another limitation was that neither study excluded patients with biliary tract reconstructions [30,31].

Evaluating hospital costs is an essential factor in value-based care [32]. Healthcare resources are limited, so determining the most cost-effective method is important [33]. Various parenchymal transection techniques have been criticized for their high costs [34,35]. In our study, the ICU stay was 1.9 days shorter in the hybrid group (5.7 days in the hybrid group versus 7.6 days in the stapler group), resulting in a 37% reduction in ICU costs (EUR 7269 in the hybrid group versus EUR 9979 in the stapler group). Additionally, patients in the hybrid group had a hospital stay that was 4.2 days shorter than in the stapler group (14.9 days in the hybrid group versus 19.1 days in the stapler group), leading to a 30% reduction in ward costs (EUR 4677 in the hybrid group versus EUR 6044 in the stapler group). The demand for ICU and hospital capacity is increasing in most healthcare centers, making it important to use surgical methods that reduce postoperative complications and minimize postoperative admissions [36]. In our study, less bile leakage in the hybrid technique contributed to this goal. Patients who underwent resection with the hybrid method also had a positive financial difference more than two-fold greater (a difference of EUR 2627.7 between the groups: EUR 4898 in the hybrid group versus EUR 2271 in the stapler group), which is particularly significant in high-volume referral centers like ours. Multiplying the calculated difference by the number of patients who underwent major liver resection in our study results in approximately EUR one million, a financially relevant difference for the healthcare system. Therefore, this finding suggests that the hybrid technique saves both money and resources.

Although this was not a randomized controlled trial and did not have the advantage of reduced bias, this high-volume matched study has some advantages over our earlier investigations. First, our study population was larger, allowing us to detect significant differences in postoperative morbidities between groups. Furthermore, we only included patients undergoing major liver resections, which are associated with higher complication rates [5] and are more influenced by surgical techniques and instruments than minor resections. This made our study population more uniform than those in other studies, especially since patients with biliary duct reconstructions were excluded. Another advantage of our study is that we analyzed these methods not only from a clinical perspective but also from a financial standpoint.

In conclusion, the results of this large-scale study of patients undergoing major hepatectomy revealed that postoperative bile leakage, as well as ICU and hospital stays, were reduced following the hybrid technique compared with the stapler method, although operation time was shorter with the stapler method. The hybrid technique was also more cost efficient and yielded a higher positive financial difference than the stapler method. This data provides a solid foundation for population calculations in future randomized controlled trials.

## Figures and Tables

**Table 1:** Patient characteristics and preoperative factors before matching in the two study groups.

		Total (N = 492)	Hybrid (N = 152)	Stapler (N = 340)	*p* Value
**Age (year)**		57.7 ± 15.9	56.42 ± 13.5	57.98 ± 11.4	0.23 ^§^
**Gender (male)**		268 (54.7%)	76 (50.0%)	192 (56.5%)	0.183 ^‡^
**BMI**		25.7 ± 4.7	25.47 ± 4.7	25.81 ± 4.67	0.338 ^§^
**ASA**	**I**	22 (4.47%)	7 (4.6%)	15 (4.4%)	0.637 *
	**II**	240 (48.78%)	75 (49.3%)	165 (48.5%)	
	**III**	225 (45.73%)	70 (46.1%)	155 (45.6%)	
	**IV**	5 (1.01%)	0 (0%)	5 (1.5%)	
**Diagnosis**	**HCC**	48 (9.75%)	12 (7.9%)	36 (10.6%)	0.622 ^‡^
	**CCC**	91 (18.49%)	30 (19.7%)	61 (17.9%)	
	**Others**	353 (71.74%)	110 (72.4%)	243 (71.5%)	
**Preoperative chemo/radiotherapy**		219 (44.51%)	63 (41.4%)	156 (45.9%)	0.360 ^‡^

BMI: Body Mass Index, ASA: American Society of Anesthesiologists physical status classification, HCC: hepatocellular carcinoma, CCC: cholangiocarcinoma, * Fisher’s test, ^§^ Mann–Whitney U test, ^‡^ chi-square test.

**Table 2:** Patient characteristics and preoperative factors after matching in the two study groups.

		Hybrid (N = 152)	Stapler (N = 304)	*p* Value
**Age (year)**		59.2 ± 14.8	58.4 ± 14.8	0.564 ^§^
**Gender (male)**		76 (50.0%)	192 (56.5%)	0.183 ^‡^
**BMI**		25.4± 4.9	25.8 ± 4.8	0.402 ^§^
**ASA**	**I**	7 (4.6%)	15 (4.9%)	0.550 *
	**II**	75 (49.3%)	144 (47.7%)	
	**III**	70 (46.1%)	141 (46.4%)	
	**IV**	0 (0%)	4 (1.3%)	
**Diagnosis**	**HCC**	12 (7.9%)	32 (10.5%)	0.599 ^‡^
	**CCC**	30 (19.7%)	53 (17.4%)	
	**Others**	110 (72.4%)	219 (72.0%)	
**Preoperative chemo/radiotherapy**		63 (41.4%)	138 (45.4%)	0.484 ^‡^

BMI: Body Mass Index, ASA: American Society of Anesthesiologists physical status classification, HCC: hepatocellular carcinoma, CCC: cholangiocarcinoma, * Fisher’s test, ^§^ Mann–Whitney U test, ^‡^ chi-square test.

**Table 3:** Comparison of intraoperative data between the two groups.

		Hybrid (N = 152)	Stapler (N = 304)	*p* Value
**Hemihepatectomy, n (%)**	**Right (N= 296)**	87 (75.7%)	209 (75.7%)	1.000 ^‡^
	**Left (N = 95)**	28 (24.3%)	67 (24.3%)	
**Extended hepatectomy, n (%)**	**Right (N = 16)**	11 (29.7%)	5 (17.9%)	0.385 ^‡^
	**Left (N = 49)**	26 (70.3%)	23 (82.1%)	
**Blood loss, mean ± SD (mL)**		1146.2 ± 821.5	1037.6 ± 743.2	0.170 ^§^
**Blood transfusion, n (%)**		24 (15.8%)	51 (16.8%)	0.894 ^‡^
**Blood transfusion, mean ± SD (unit)**		0.66 ± 2.59	0.33 ± 1.01	0.260 ^‡^
**Operation time, mean ± SD**		224.8 ± 83.7	202.6 ± 67.6	**0.005** ^§^
**Number of the used stapler, median (range)**		4 (3–6)	11 (8–13)	**<0.001** ^⊥^

SD: Standard Deviation, ^§^ independent *t*-test, ^‡^ chi-square test, ^⊥^ Mann–Whitney U test.

**Table 4:** Comparison of postoperative data between the two groups.

	Hybrid (N = 152)	Stapler (N = 304)	*p* Value
**Bile leakage, N (%)**	20 (13.2%)	78 (25.7%)	**0.002** ^‡^
**Bleeding, N (%)**	1 (0.7%)	12 (3.9%)	0.069 ^‡^
**Relaparotomy, N (%)**	18 (11.8%)	36 (11.8%)	0.566 ^‡^
Major complications, N (%) *	45 (29.6%)	107 (35.2%)	0.248 ^‡^
**ICU stay, mean ± SD (days)**	5.7 ± 5.3	7.6 ± 5.9	**0.034 ^§^**
**Hospital stay, mean ± SD (days)**	14.9 ± 12.7	19.1 ± 17.2	**0.007** ^§^
**Mortality, N (%)**	8 (5.3%)	12 (3.9%)	0.628 ^‡^

ICU: intensive care unit, SD: Standard Deviation, ^§^ independent *t*-test, ^‡^ chi-square test, * patients may have more than one complication.

**Table 5:** Cost comparison between the two groups.

	Hybrid (N = 152) (Mean ± SE)	Stapler (N = 304) (Mean ± SE)	*p* Value
**Surgical devices costs (EUR)**	1149.9 ± 6.6	1464.9 ± 6.1	**<0.001** ^§^
**Operating room costs (EUR)**	7417.6 ± 224.1	6663.8 ± 129.6	**0.004** ^§^
**Total operative costs (EUR)**	8567.6 ± 224.9	8128.8 ± 128.6	0.092 ^§^
**Total ICU costs** * **(EUR)**	7269.3 ± 760.9	9979.4 ± 918.2	**0.024** ^§^
**Total ward costs (EUR)**	4677.1 ± 385.8	6044.2 ± 341.5	**0.014 ^§^**
**Total hospital costs (EUR)**	18,170.6 ± 737.1	20,245.9 ± 768.2	0.052 ^§^
**Diagnosis-related reimbursement (EUR)**	23,377.1 ± 1634.7	22,666.4 ± 1272.2	0.740 ^§^
Financial difference ** (EUR)	4898.9 ± 1532.7	2271.2 ± 1159.8	**0.020 ^§^**

ICU: intensive care unit, SE: Standard Error, ^§^ independent *t*-test. * This was calculated only for patients who had postoperative ICU stays. ** This was calculated as diagnosis-related reimbursement—total hospital costs.

## Data Availability

Anonymized data supporting the findings of this study are available from the corresponding author upon request, after approval by the ethics committee. This is due to privacy and ethical restrictions.

## Supplementary Materials

The following supporting information can be downloaded at https://www.mdpi.com/article/10.3390/jcm14113892/s1, Table S1: Comparison of postoperative outcomes between the hybrid and stapler groups before propensity score matching.
