# Supplementary material for: Outcomes and Cost of Major Liver Resection Using Combined LigaSure and Stapler: A Propensity Score Matching Study
Source: J Clin Med. 2025 Jun 1;14(11):3892. doi: 10.3390/jcm14113892 (PMC12156395; doi:10.3390/jcm14113892)
Supplement: Supplementary file 1 [file jcm-14-03892-s001.zip › jcm-3641790-supplementary.pdf]

Table S1. Comparison of postoperative outcomes between the hybrid and stapler groups before propensity score matching.

	Hybrid (N=152)	Stapler (N=340)	*P value*
**Bile leakage, N (%)**	20 (13.2%)	87 (25.6%)	**0.002**‡
**Bleeding, N (%)**	1 (0.7%)	17 (5.0%)	**0.017**‡
**Relaparotomy, N (%)**	18 (11.8%)	46 (13.5%)	0.607‡
**Major complications, N (%)***	45 (29.6%)	126 (37.1%)	0.109‡
**ICU stay, mean ± SD (days)**	5.7 ± 5.3	7.3 ± 6.5	**0.041**§
**Hospital stay, mean ± SD (days)**	14.9 ± 12.7	15.09 ± 15.44	0.619§
**Mortality, N (%)**	8 (5.3%)	17 (5.0%)	0.902‡

ICU: Intensive Care Unit, SD: Standard, § Independent t-test, ‡ Chi-square test, * Patients may have more than one complication
